# Children’s Disorders among the Japanese

**Published:** 1878-02

**Authors:** 


### Children's Disorders among the Japanese.

In regard to the personal habits of the people, it is interesting to remark that they drink very little cold water. The water is drunk as hot tea—in other words, it is boiled. Of extreme importance, too, in regard to children's disorders, is the fact that, until they are two or three years old, they draw their nourishment from the maternal fount. *No child is fed artificially.*

On the other hand, it is interesting to note that the Japanese eat unripe fruit to an inordinate extent. The moment fruit shows the slightest signs of being soft as an evidence of ripeness, it is considered by them as unfit to eat. It is astonishing to see them eat hard, green peaches—clinching them in the fist, as a country boy does a hard apple, and biting off each mouthful with a loud snap. They eat their pears in the same way; cucumbers are eaten in a more unripe condition than with us even; and water-melons, which are so much inveighed against at home, are here eaten by all classes and at all times.

In fact, they seem to revel in those things which at home are considered so productive of summer-complaints; who does not recall the astonishment he has felt at the sight of country children of tender age eating green apples, green corn uncooked, and similar things, and yet suffering no ill-effects therefrom? These facts may not prove, perhaps, that unripe fruit is harmless; but, in connection with the other statements, they do show that the removal of sewage-matter from houses is the important point to consider, and that its removal insures an absence, or a less number, of cases of those diseases which enhance our death-rate at home, and lends an additional reason for the necessity of vigilance on the part of communities regarding these matters.—*Popular Science Monthly.*

